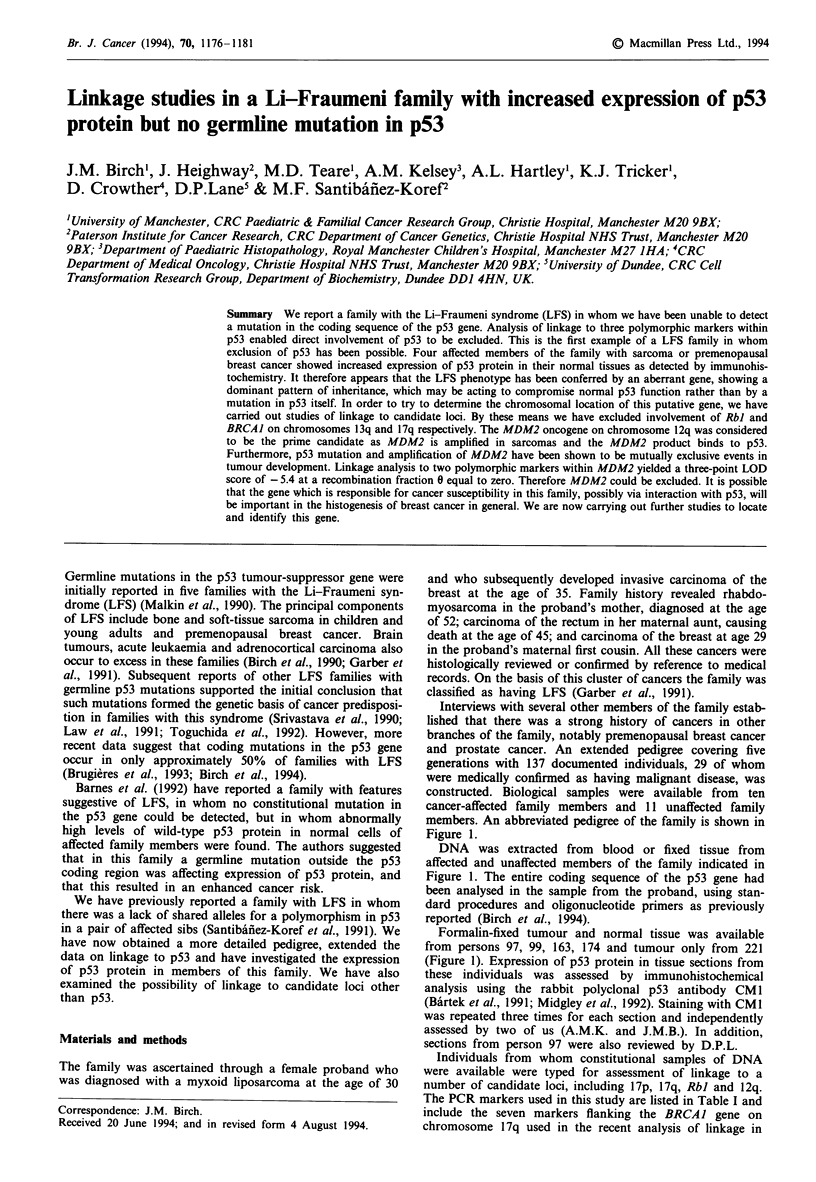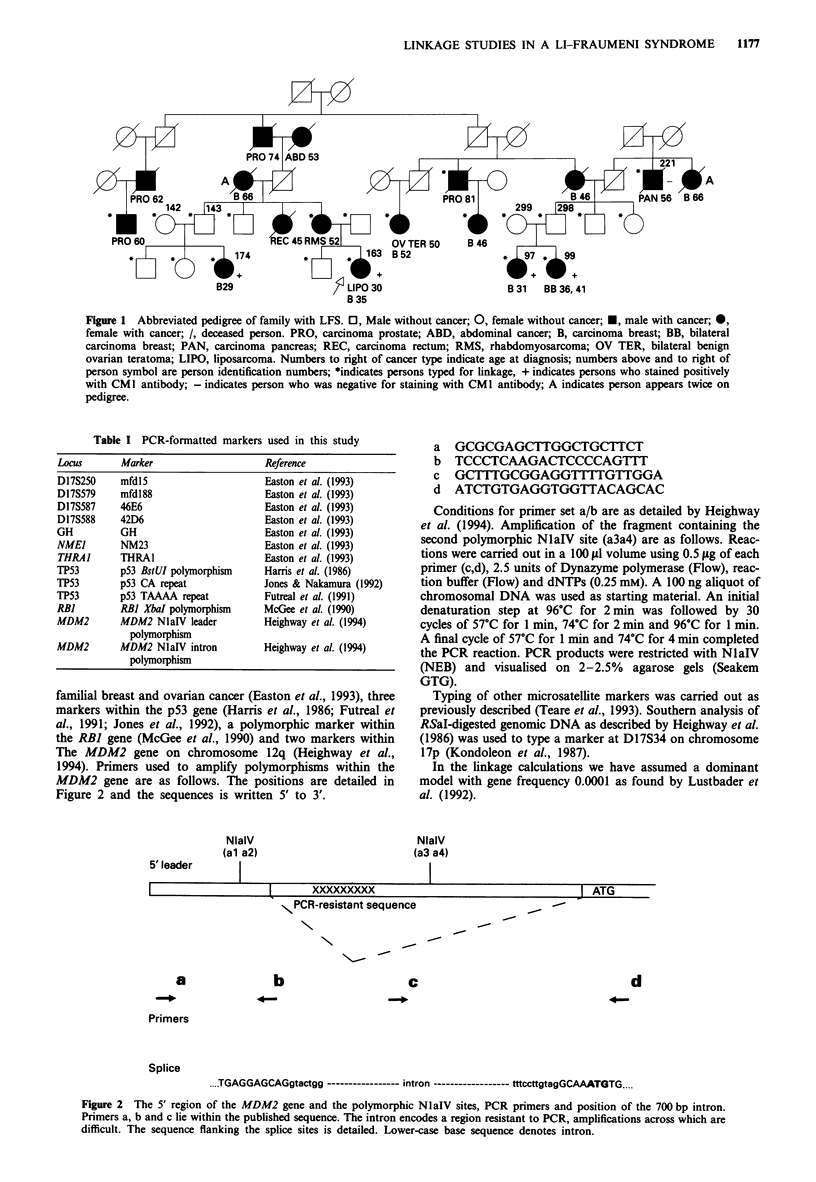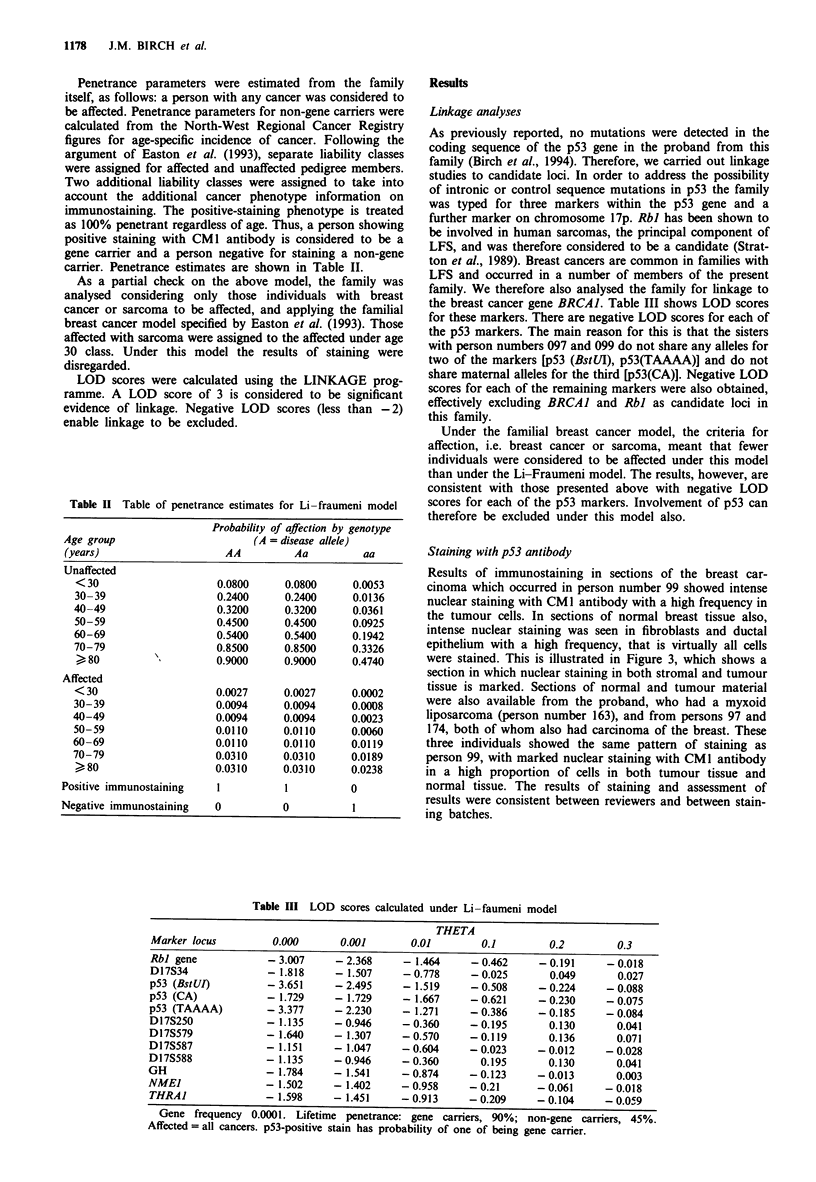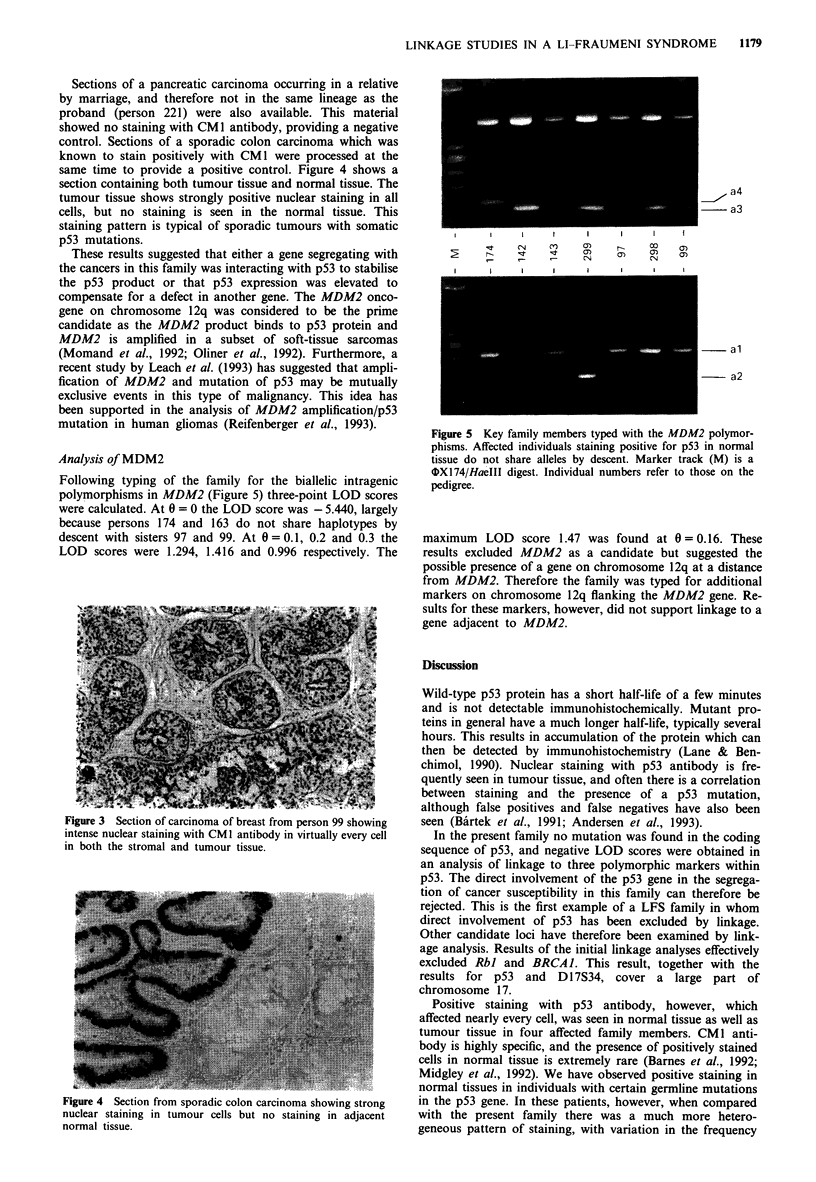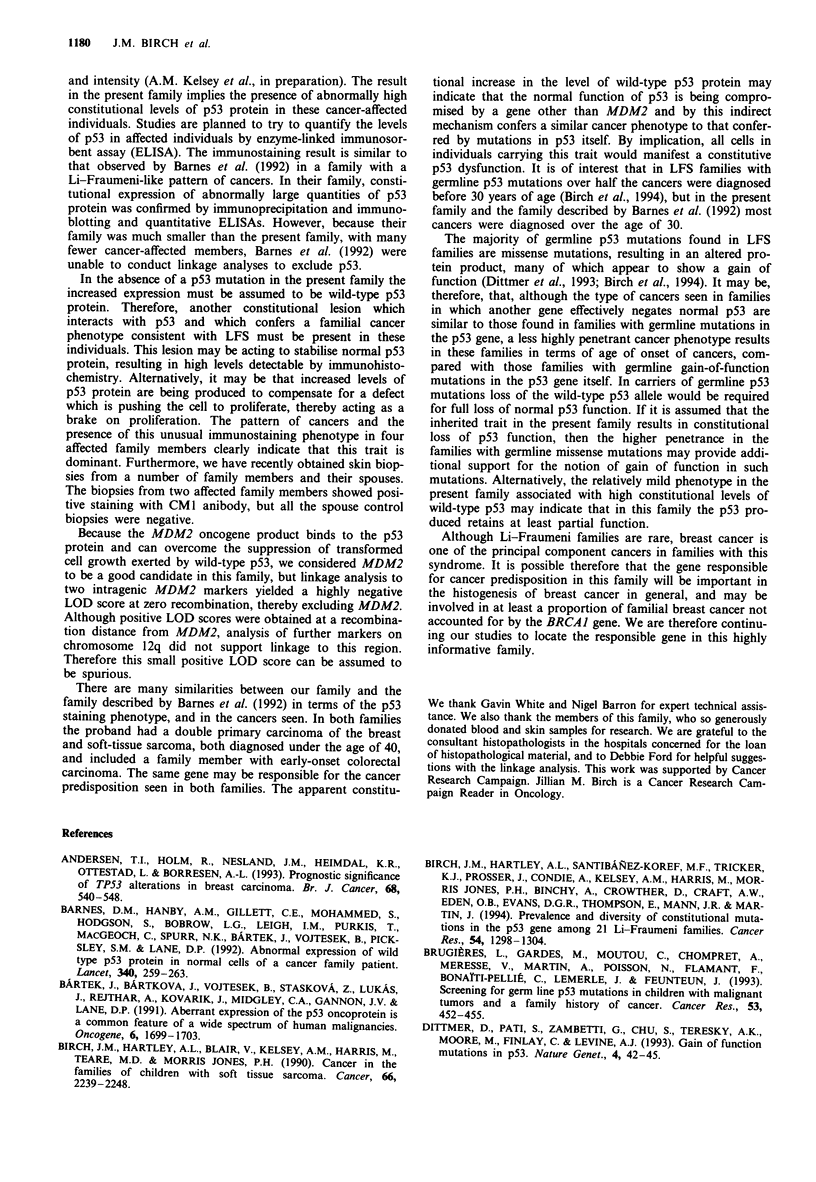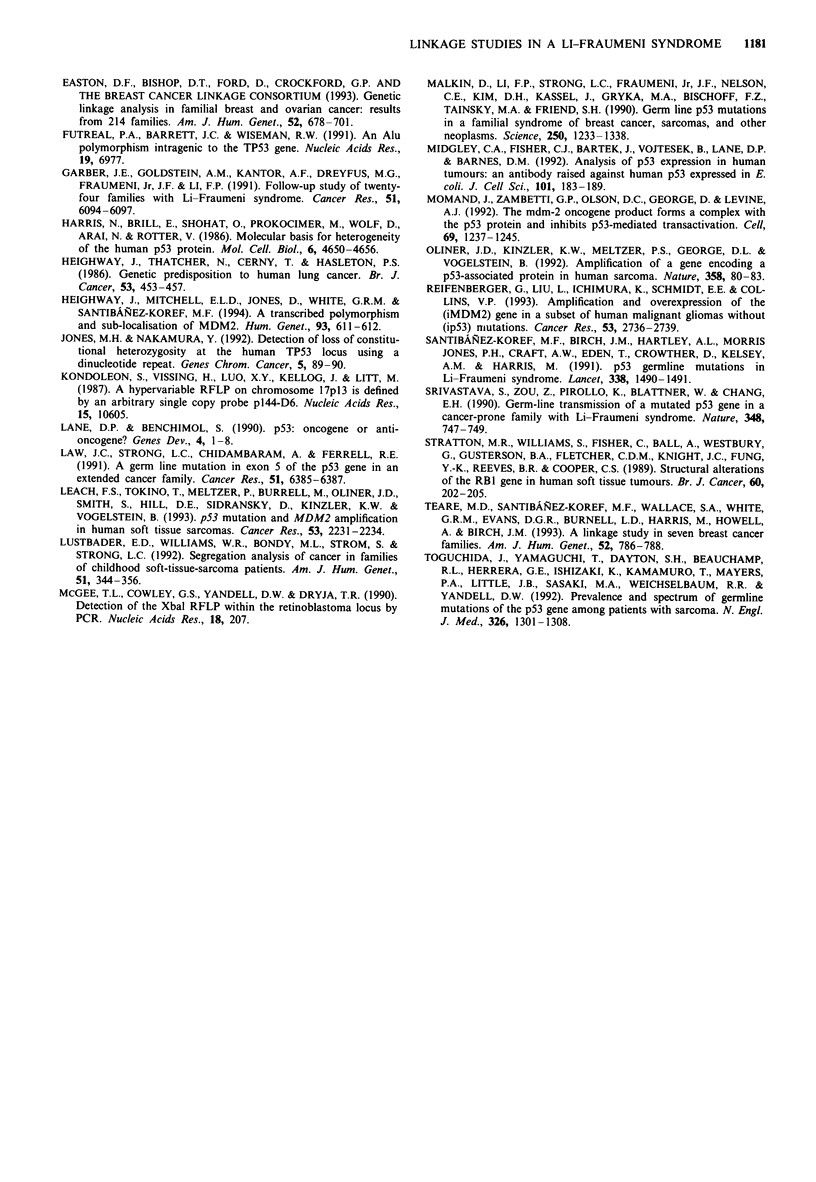# Linkage studies in a Li-Fraumeni family with increased expression of p53 protein but no germline mutation in p53.

**DOI:** 10.1038/bjc.1994.468

**Published:** 1994-12

**Authors:** J. M. Birch, J. Heighway, M. D. Teare, A. M. Kelsey, A. L. Hartley, K. J. Tricker, D. Crowther, D. P. Lane, M. F. Santibáñez-Koref

**Affiliations:** University of Manchester, CRC Paediatric & Familial Cancer Research Group, Christie Hospital, UK.

## Abstract

**Images:**


Br. J. Cancer (1994), 70, 1176-1181 © Macmillan Press Ltd., 1994

### Linkage studies in a Li-Fraumeni family with increased expression of p53 protein but no germline mutation in p53

J.M. Birch[1], J. Heighway[2], M.D. Teare[1], A.M. Kelsey[3], A.L. Hartley[1], K.J. Tricker[1], D. Crowther[4], D.P.Lane[5] & M.F. Santibáñez-Koref[2]

[1]*University of Manchester, CRC Paediatric & Familial Cancer Research Group, Christie Hospital, Manchester M20 9BX;* [2]*Paterson Institute for Cancer Research, CRC Department of Cancer Genetics, Christie Hospital NHS Trust, Manchester M20 9BX;* [3]*Department of Paediatric Histopathology, Royal Manchester Children's Hospital, Manchester M27 1HA;* [4]*CRC Department of Medical Oncology, Christie Hospital NHS Trust, Manchester M20 9BX;* [5]*University of Dundee, CRC Cell Transformation Research Group, Department of Biochemistry, Dundee DD1 4HN, UK.*

**Summary** We report a family with the Li-Fraumeni syndrome (LFS) in whom we have been unable to detect a mutation in the coding sequence of the p53 gene. Analysis of linkage to three polymorphic markers within p53 enabled direct involvement of p53 to be excluded. This is the first example of a LFS family in whom exclusion of p53 has been possible. Four affected members of the family with sarcoma or premenopausal breast cancer showed increased expression of p53 protein in their normal tissues as detected by immunohistochemistry. It therefore appears that the LFS phenotype has been conferred by an aberrant gene, showing a dominant pattern of inheritance, which may be acting to compromise normal p53 function rather than by a mutation in p53 itself. In order to try to determine the chromosomal location of this putative gene, we have carried out studies of linkage to candidate loci. By these means we have excluded involvement of *Rb1* and *BRCA1* on chromosomes 13q and 17q respectively. The *MDM2* oncogene on chromosome 12q was considered to be the prime candidate as *MDM2* is amplified in sarcomas and the *MDM2* product binds to p53. Furthermore, p53 mutation and amplification of *MDM2* have been shown to be mutually exclusive events in tumour development. Linkage analysis to two polymorphic markers within *MDM2* yielded a three-point LOD score of $-5.4$ at a recombination fraction $\theta$ equal to zero. Therefore *MDM2* could be excluded. It is possible that the gene which is responsible for cancer susceptibility in this family, possibly via interaction with p53, will be important in the histogenesis of breast cancer in general. We are now carrying out further studies to locate and identify this gene.

Germline mutations in the p53 tumour-suppressor gene were initially reported in five families with the Li-Fraumeni syndrome (LFS) (Malkin *et al.*, 1990). The principal components of LFS include bone and soft-tissue sarcoma in children and young adults and premenopausal breast cancer. Brain tumours, acute leukaemia and adrenocortical carcinoma also occur to excess in these families (Birch *et al.*, 1990; Garber *et al.*, 1991). Subsequent reports of other LFS families with germline p53 mutations supported the initial conclusion that such mutations formed the genetic basis of cancer predisposition in families with this syndrome (Srivastava *et al.*, 1990; Law *et al.*, 1991; Toguchida *et al.*, 1992). However, more recent data suggest that coding mutations in the p53 gene occur in only approximately 50% of families with LFS (Brugières *et al.*, 1993; Birch *et al.*, 1994).

Barnes *et al.* (1992) have reported a family with features suggestive of LFS, in whom no constitutional mutation in the p53 gene could be detected, but in whom abnormally high levels of wild-type p53 protein in normal cells of affected family members were found. The authors suggested that in this family a germline mutation outside the p53 coding region was affecting expression of p53 protein, and that this resulted in an enhanced cancer risk.

We have previously reported a family with LFS in whom there was a lack of shared alleles for a polymorphism in p53 in a pair of affected sibs (Santibáñez-Koref *et al.*, 1991). We have now obtained a more detailed pedigree, extended the data on linkage to p53 and have investigated the expression of p53 protein in members of this family. We have also examined the possibility of linkage to candidate loci other than p53.

### Materials and methods

The family was ascertained through a female proband who was diagnosed with a myxoid liposarcoma at the age of 30 and who subsequently developed invasive carcinoma of the breast at the age of 35. Family history revealed rhabdomyosarcoma in the proband's mother, diagnosed at the age of 52; carcinoma of the rectum in her maternal aunt, causing death at the age of 45; and carcinoma of the breast at age 29 in the proband's maternal first cousin. All these cancers were histologically reviewed or confirmed by reference to medical records. On the basis of this cluster of cancers the family was classified as having LFS (Garber *et al.*, 1991).

Interviews with several other members of the family established that there was a strong history of cancers in other branches of the family, notably premenopausal breast cancer and prostate cancer. An extended pedigree covering five generations with 137 documented individuals, 29 of whom were medically confirmed as having malignant disease, was constructed. Biological samples were available from ten cancer-affected family members and 11 unaffected family members. An abbreviated pedigree of the family is shown in Figure 1.

DNA was extracted from blood or fixed tissue from affected and unaffected members of the family indicated in Figure 1. The entire coding sequence of the p53 gene had been analysed in the sample from the proband, using standard procedures and oligonucleotide primers as previously reported (Birch *et al.*, 1994).

Formalin-fixed tumour and normal tissue was available from persons 97, 99, 163, 174 and tumour only from 221 (Figure 1). Expression of p53 protein in tissue sections from these individuals was assessed by immunohistochemical analysis using the rabbit polyclonal p53 antibody CM1 (Bártek *et al.*, 1991; Midgley *et al.*, 1992). Staining with CM1 was repeated three times for each section and independently assessed by two of us (A.M.K. and J.M.B.). In addition, sections from person 97 were also reviewed by D.P.L.

Individuals from whom constitutional samples of DNA were available were typed for assessment of linkage to a number of candidate loci, including 17p, 17q, *Rb1* and 12q. The PCR markers used in this study are listed in Table I and include the seven markers flanking the *BRCA1* gene on chromosome 17q used in the recent analysis of linkage in

Correspondence: J.M. Birch.
Received 20 June 1994; and in revised form 4 August 1994.

**Figure 1** Abbreviated pedigree of family with LFS. □, Male without cancer; ○, female without cancer; ■, male with cancer; ●, female with cancer; /, deceased person. PRO, carcinoma prostate; ABD, abdominal cancer; B, carcinoma breast; BB, bilateral carcinoma breast; PAN, carcinoma pancreas; REC, carcinoma rectum; RMS, rhabdomyosarcoma; OV TER, bilateral benign ovarian teratoma; LIPO, liposarcoma. Numbers to right of cancer type indicate age at diagnosis; numbers above and to right of person symbol are person identification numbers; *indicates persons typed for linkage, + indicates persons who stained positively with CM1 antibody; – indicates person who was negative for staining with CM1 antibody; A indicates person appears twice on pedigree.

**Table I** PCR-formatted markers used in this study

| *Locus* | *Marker* | *Reference* |
|---|---|---|
| D17S250 | mfd15 | Easton *et al.* (1993) |
| D17S579 | mfd188 | Easton *et al.* (1993) |
| D17S587 | 46E6 | Easton *et al.* (1993) |
| D17S588 | 42D6 | Easton *et al.* (1993) |
| GH | GH | Easton *et al.* (1993) |
| *NME1* | NM23 | Easton *et al.* (1993) |
| *THRA1* | THRA1 | Easton *et al.* (1993) |
| TP53 | p53 *BstUI* polymorphism | Harris *et al.* (1986) |
| TP53 | p53 CA repeat | Jones & Nakamura (1992) |
| TP53 | p53 TAAAA repeat | Futreal *et al.* (1991) |
| *RB1* | *RB1 XbaI* polymorphism | McGee *et al.* (1990) |
| *MDM2* | *MDM2* NlaIV leader polymorphism | Heighway *et al.* (1994) |
| *MDM2* | *MDM2* NlaIV intron polymorphism | Heighway *et al.* (1994) |

familial breast and ovarian cancer (Easton *et al.*, 1993), three markers within the p53 gene (Harris *et al.*, 1986; Futreal *et al.*, 1991; Jones *et al.*, 1992), a polymorphic marker within the *RB1* gene (McGee *et al.*, 1990) and two markers within The *MDM2* gene on chromosome 12q (Heighway *et al.*, 1994). Primers used to amplify polymorphisms within the *MDM2* gene are as follows. The positions are detailed in Figure 2 and the sequences is written 5′ to 3′.

a GCGCGAGCTTGGCTGCTTCT
b TCCCTCAAGACTCCCCAGTTT
c GCTTTGCGGAGGTTTTGTTGGA
d ATCTGTGAGGTGGTTACAGCAC

Conditions for primer set a/b are as detailed by Heighway *et al.* (1994). Amplification of the fragment containing the second polymorphic NlaIV site (a3a4) are as follows. Reactions were carried out in a 100 µl volume using 0.5 µg of each primer (c,d), 2.5 units of Dynazyme polymerase (Flow), reaction buffer (Flow) and dNTPs (0.25 mM). A 100 ng aliquot of chromosomal DNA was used as starting material. An initial denaturation step at 96°C for 2 min was followed by 30 cycles of 57°C for 1 min, 74°C for 2 min and 96°C for 1 min. A final cycle of 57°C for 1 min and 74°C for 4 min completed the PCR reaction. PCR products were restricted with NlaIV (NEB) and visualised on 2–2.5% agarose gels (Seakem GTG).

Typing of other microsatellite markers was carried out as previously described (Teare *et al.*, 1993). Southern analysis of *RSa*I-digested genomic DNA as described by Heighway *et al.* (1986) was used to type a marker at D17S34 on chromosome 17p (Kondoleon *et al.*, 1987).

In the linkage calculations we have assumed a dominant model with gene frequency 0.0001 as found by Lustbader *et al.* (1992).

**Figure 2** The 5′ region of the *MDM2* gene and the polymorphic NlaIV sites, PCR primers and position of the 700 bp intron. Primers a, b and c lie within the published sequence. The intron encodes a region resistant to PCR, amplifications across which are difficult. The sequence flanking the splice sites is detailed. Lower-case base sequence denotes intron.

Penetrance parameters were estimated from the family itself, as follows: a person with any cancer was considered to be affected. Penetrance parameters for non-gene carriers were calculated from the North-West Regional Cancer Registry figures for age-specific incidence of cancer. Following the argument of Easton et al. (1993), separate liability classes were assigned for affected and unaffected pedigree members. Two additional liability classes were assigned to take into account the additional cancer phenotype information on immunostaining. The positive-staining phenotype is treated as 100% penetrant regardless of age. Thus, a person showing positive staining with CM1 antibody is considered to be a gene carrier and a person negative for staining a non-gene carrier. Penetrance estimates are shown in Table II.

As a partial check on the above model, the family was analysed considering only those individuals with breast cancer or sarcoma to be affected, and applying the familial breast cancer model specified by Easton et al. (1993). Those affected with sarcoma were assigned to the affected under age 30 class. Under this model the results of staining were disregarded.

LOD scores were calculated using the LINKAGE programme. A LOD score of 3 is considered to be significant evidence of linkage. Negative LOD scores (less than −2) enable linkage to be excluded.

**Table II** Table of penetrance estimates for Li-fraumeni model

| Age group (years) | Probability of affection by genotype (A = disease allele) AA | Aa | aa |
|---|---|---|---|
| Unaffected | | | |
| <30 | 0.0800 | 0.0800 | 0.0053 |
| 30-39 | 0.2400 | 0.2400 | 0.0136 |
| 40-49 | 0.3200 | 0.3200 | 0.0361 |
| 50-59 | 0.4500 | 0.4500 | 0.0925 |
| 60-69 | 0.5400 | 0.5400 | 0.1942 |
| 70-79 | 0.8500 | 0.8500 | 0.3326 |
| ⩾80 | 0.9000 | 0.9000 | 0.4740 |
| Affected | | | |
| <30 | 0.0027 | 0.0027 | 0.0002 |
| 30-39 | 0.0094 | 0.0094 | 0.0008 |
| 40-49 | 0.0094 | 0.0094 | 0.0023 |
| 50-59 | 0.0110 | 0.0110 | 0.0060 |
| 60-69 | 0.0110 | 0.0110 | 0.0119 |
| 70-79 | 0.0310 | 0.0310 | 0.0189 |
| ⩾80 | 0.0310 | 0.0310 | 0.0238 |
| Positive immunostaining | 1 | 1 | 0 |
| Negative immunostaining | 0 | 0 | 1 |

### Results

### Linkage analyses

As previously reported, no mutations were detected in the coding sequence of the p53 gene in the proband from this family (Birch et al., 1994). Therefore, we carried out linkage studies to candidate loci. In order to address the possibility of intronic or control sequence mutations in p53 the family was typed for three markers within the p53 gene and a further marker on chromosome 17p. *Rb1* has been shown to be involved in human sarcomas, the principal component of LFS, and was therefore considered to be a candidate (Stratton et al., 1989). Breast cancers are common in families with LFS and occurred in a number of members of the present family. We therefore also analysed the family for linkage to the breast cancer gene *BRCA1*. Table III shows LOD scores for these markers. There are negative LOD scores for each of the p53 markers. The main reason for this is that the sisters with person numbers 097 and 099 do not share any alleles for two of the markers [p53 (*BstUI*), p53(TAAAA)] and do not share maternal alleles for the third [p53(CA)]. Negative LOD scores for each of the remaining markers were also obtained, effectively excluding *BRCA1* and *Rb1* as candidate loci in this family.

Under the familial breast cancer model, the criteria for affection, i.e. breast cancer or sarcoma, meant that fewer individuals were considered to be affected under this model than under the Li-Fraumeni model. The results, however, are consistent with those presented above with negative LOD scores for each of the p53 markers. Involvement of p53 can therefore be excluded under this model also.

### Staining with p53 antibody

Results of immunostaining in sections of the breast carcinoma which occurred in person number 99 showed intense nuclear staining with CM1 antibody with a high frequency in the tumour cells. In sections of normal breast tissue also, intense nuclear staining was seen in fibroblasts and ductal epithelium with a high frequency, that is virtually all cells were stained. This is illustrated in Figure 3, which shows a section in which nuclear staining in both stromal and tumour tissue is marked. Sections of normal and tumour material were also available from the proband, who had a myxoid liposarcoma (person number 163), and from persons 97 and 174, both of whom also had carcinoma of the breast. These three individuals showed the same pattern of staining as person 99, with marked nuclear staining with CM1 antibody in a high proportion of cells in both tumour tissue and normal tissue. The results of staining and assessment of results were consistent between reviewers and between staining batches.

**Table III** LOD scores calculated under Li-faumeni model

| Marker locus | THETA 0.000 | 0.001 | 0.01 | 0.1 | 0.2 | 0.3 |
|---|---|---|---|---|---|---|
| *Rb1* gene | −3.007 | −2.368 | −1.464 | −0.462 | −0.191 | −0.018 |
| D17S34 | −1.818 | −1.507 | −0.778 | −0.025 | 0.049 | 0.027 |
| p53 (*BstUI*) | −3.651 | −2.495 | −1.519 | −0.508 | −0.224 | −0.088 |
| p53 (CA) | −1.729 | −1.729 | −1.667 | −0.621 | −0.230 | −0.075 |
| p53 (TAAAA) | −3.377 | −2.230 | −1.271 | −0.386 | −0.185 | −0.084 |
| D17S250 | −1.135 | −0.946 | −0.360 | −0.195 | 0.130 | 0.041 |
| D17S579 | −1.640 | −1.307 | −0.570 | −0.119 | 0.136 | 0.071 |
| D17S587 | −1.151 | −1.047 | −0.604 | −0.023 | −0.012 | −0.028 |
| D17S588 | −1.135 | −0.946 | −0.360 | 0.195 | 0.130 | 0.041 |
| GH | −1.784 | −1.541 | −0.874 | −0.123 | −0.013 | 0.003 |
| *NME1* | −1.502 | −1.402 | −0.958 | −0.21 | −0.061 | −0.018 |
| *THRA1* | −1.598 | −1.451 | −0.913 | −0.209 | −0.104 | −0.059 |

Gene frequency 0.0001. Lifetime penetrance: gene carriers, 90%; non-gene carriers, 45%. Affected = all cancers. p53-positive stain has probability of one of being gene carrier.

Sections of a pancreatic carcinoma occurring in a relative by marriage, and therefore not in the same lineage as the proband (person 221) were also available. This material showed no staining with CM1 antibody, providing a negative control. Sections of a sporadic colon carcinoma which was known to stain positively with CM1 were processed at the same time to provide a positive control. Figure 4 shows a section containing both tumour tissue and normal tissue. The tumour tissue shows strongly positive nuclear staining in all cells, but no staining is seen in the normal tissue. This staining pattern is typical of sporadic tumours with somatic p53 mutations.

These results suggested that either a gene segregating with the cancers in this family was interacting with p53 to stabilise the p53 product or that p53 expression was elevated to compensate for a defect in another gene. The *MDM2* oncogene on chromosome 12q was considered to be the prime candidate as the *MDM2* product binds to p53 protein and *MDM2* is amplified in a subset of soft-tissue sarcomas (Momand *et al.*, 1992; Oliner *et al.*, 1992). Furthermore, a recent study by Leach *et al.* (1993) has suggested that amplification of *MDM2* and mutation of p53 may be mutually exclusive events in this type of malignancy. This idea has been supported in the analysis of *MDM2* amplification/p53 mutation in human gliomas (Reifenberger *et al.*, 1993).

### *Analysis of* MDM2

Following typing of the family for the biallelic intragenic polymorphisms in *MDM2* (Figure 5) three-point LOD scores were calculated. At $\theta = 0$ the LOD score was $-5.440$, largely because persons 174 and 163 do not share haplotypes by descent with sisters 97 and 99. At $\theta = 0.1$, 0.2 and 0.3 the LOD scores were 1.294, 1.416 and 0.996 respectively. The maximum LOD score 1.47 was found at $\theta = 0.16$. These results excluded *MDM2* as a candidate but suggested the possible presence of a gene on chromosome 12q at a distance from *MDM2*. Therefore the family was typed for additional markers on chromosome 12q flanking the *MDM2* gene. Results for these markers, however, did not support linkage to a gene adjacent to *MDM2*.

**Figure 3** Section of carcinoma of breast from person 99 showing intense nuclear staining with CM1 antibody in virtually every cell in both the stromal and tumour tissue.

**Figure 4** Section from sporadic colon carcinoma showing strong nuclear staining in tumour cells but no staining in adjacent normal tissue.

**Figure 5** Key family members typed with the *MDM2* polymorphisms. Affected individuals staining positive for p53 in normal tissue do not share alleles by descent. Marker track (M) is a ΦX174/*Hae*III digest. Individual numbers refer to those on the pedigree.

### Discussion

Wild-type p53 protein has a short half-life of a few minutes and is not detectable immunohistochemically. Mutant proteins in general have a much longer half-life, typically several hours. This results in accumulation of the protein which can then be detected by immunohistochemistry (Lane & Benchimol, 1990). Nuclear staining with p53 antibody is frequently seen in tumour tissue, and often there is a correlation between staining and the presence of a p53 mutation, although false positives and false negatives have also been seen (Bártek *et al.*, 1991; Andersen *et al.*, 1993).

In the present family no mutation was found in the coding sequence of p53, and negative LOD scores were obtained in an analysis of linkage to three polymorphic markers within p53. The direct involvement of the p53 gene in the segregation of cancer susceptibility in this family can therefore be rejected. This is the first example of a LFS family in whom direct involvement of p53 has been excluded by linkage. Other candidate loci have therefore been examined by linkage analysis. Results of the initial linkage analyses effectively excluded *Rb1* and *BRCA1*. This result, together with the results for p53 and D17S34, cover a large part of chromosome 17.

Positive staining with p53 antibody, however, which affected nearly every cell, was seen in normal tissue as well as tumour tissue in four affected family members. CM1 antibody is highly specific, and the presence of positively stained cells in normal tissue is extremely rare (Barnes *et al.*, 1992; Midgley *et al.*, 1992). We have observed positive staining in normal tissues in individuals with certain germline mutations in the p53 gene. In these patients, however, when compared with the present family there was a much more heterogeneous pattern of staining, with variation in the frequency and intensity (A.M. Kelsey *et al.*, in preparation). The result in the present family implies the presence of abnormally high constitutional levels of p53 protein in these cancer-affected individuals. Studies are planned to try to quantify the levels of p53 in affected individuals by enzyme-linked immunosorbent assay (ELISA). The immunostaining result is similar to that observed by Barnes *et al.* (1992) in a family with a Li–Fraumeni-like pattern of cancers. In their family, constitutional expression of abnormally large quantities of p53 protein was confirmed by immunoprecipitation and immunoblotting and quantitative ELISAs. However, because their family was much smaller than the present family, with many fewer cancer-affected members, Barnes *et al.* (1992) were unable to conduct linkage analyses to exclude p53.

In the absence of a p53 mutation in the present family the increased expression must be assumed to be wild-type p53 protein. Therefore, another constitutional lesion which interacts with p53 and which confers a familial cancer phenotype consistent with LFS must be present in these individuals. This lesion may be acting to stabilise normal p53 protein, resulting in high levels detectable by immunohistochemistry. Alternatively, it may be that increased levels of p53 protein are being produced to compensate for a defect which is pushing the cell to proliferate, thereby acting as a brake on proliferation. The pattern of cancers and the presence of this unusual immunostaining phenotype in four affected family members clearly indicate that this trait is dominant. Furthermore, we have recently obtained skin biopsies from a number of family members and their spouses. The biopsies from two affected family members showed positive staining with CM1 anibody, but all the spouse control biopsies were negative.

Because the *MDM2* oncogene product binds to the p53 protein and can overcome the suppression of transformed cell growth exerted by wild-type p53, we considered *MDM2* to be a good candidate in this family, but linkage analysis to two intragenic *MDM2* markers yielded a highly negative LOD score at zero recombination, thereby excluding *MDM2*. Although positive LOD scores were obtained at a recombination distance from *MDM2*, analysis of further markers on chromosome 12q did not support linkage to this region. Therefore this small positive LOD score can be assumed to be spurious.

There are many similarities between our family and the family described by Barnes *et al.* (1992) in terms of the p53 staining phenotype, and in the cancers seen. In both families the proband had a double primary carcinoma of the breast and soft-tissue sarcoma, both diagnosed under the age of 40, and included a family member with early-onset colorectal carcinoma. The same gene may be responsible for the cancer predisposition seen in both families. The apparent constitutional increase in the level of wild-type p53 protein may indicate that the normal function of p53 is being compromised by a gene other than *MDM2* and by this indirect mechanism confers a similar cancer phenotype to that conferred by mutations in p53 itself. By implication, all cells in individuals carrying this trait would manifest a constitutive p53 dysfunction. It is of interest that in LFS families with germline p53 mutations over half the cancers were diagnosed before 30 years of age (Birch *et al.*, 1994), but in the present family and the family described by Barnes *et al.* (1992) most cancers were diagnosed over the age of 30.

The majority of germline p53 mutations found in LFS families are missense mutations, resulting in an altered protein product, many of which appear to show a gain of function (Dittmer *et al.*, 1993; Birch *et al.*, 1994). It may be, therefore, that, although the type of cancers seen in families in which another gene effectively negates normal p53 are similar to those found in families with germline mutations in the p53 gene, a less highly penetrant cancer phenotype results in these families in terms of age of onset of cancers, compared with those families with germline gain-of-function mutations in the p53 gene itself. In carriers of germline p53 mutations loss of the wild-type p53 allele would be required for full loss of normal p53 function. If it is assumed that the inherited trait in the present family results in constitutional loss of p53 function, then the higher penetrance in the families with germline missense mutations may provide additional support for the notion of gain of function in such mutations. Alternatively, the relatively mild phenotype in the present family associated with high constitutional levels of wild-type p53 may indicate that in this family the p53 produced retains at least partial function.

Although Li–Fraumeni families are rare, breast cancer is one of the principal component cancers in families with this syndrome. It is possible therefore that the gene responsible for cancer predisposition in this family will be important in the histogenesis of breast cancer in general, and may be involved in at least a proportion of familial breast cancer not accounted for by the *BRCA1* gene. We are therefore continuing our studies to locate the responsible gene in this highly informative family.

We thank Gavin White and Nigel Barron for expert technical assistance. We also thank the members of this family, who so generously donated blood and skin samples for research. We are grateful to the consultant histopathologists in the hospitals concerned for the loan of histopathological material, and to Debbie Ford for helpful suggestions with the linkage analysis. This work was supported by Cancer Research Campaign. Jillian M. Birch is a Cancer Research Campaign Reader in Oncology.

### References

ANDERSEN, T.I., HOLM, R., NESLAND, J.M., HEIMDAL, K.R., OTTESTAD, L. & BORRESEN, A.-L. (1993). Prognostic significance of *TP53* alterations in breast carcinoma. *Br. J. Cancer*, **68**, 540–548.

BARNES, D.M., HANBY, A.M., GILLETT, C.E., MOHAMMED, S., HODGSON, S., BOBROW, L.G., LEIGH, I.M., PURKIS, T., MACGEOCH, C., SPURR, N.K., BÁRTEK, J., VOJTESEK, B., PICKSLEY, S.M. & LANE, D.P. (1992). Abnormal expression of wild type p53 protein in normal cells of a cancer family patient. *Lancet*, **340**, 259–263.

BÁRTEK, J., BÁRTKOVA, J., VOJTESEK, B., STASKOVÁ, Z., LUKÁS, J., REJTHAR, A., KOVARIK, J., MIDGLEY, C.A., GANNON, J.V. & LANE, D.P. (1991). Aberrant expression of the p53 oncoprotein is a common feature of a wide spectrum of human malignancies. *Oncogene*, **6**, 1699–1703.

BIRCH, J.M., HARTLEY, A.L., BLAIR, V., KELSEY, A.M., HARRIS, M., TEARE, M.D. & MORRIS JONES, P.H. (1990). Cancer in the families of children with soft tissue sarcoma. *Cancer*, **66**, 2239–2248.

BIRCH, J.M., HARTLEY, A.L., SANTIBÁÑEZ-KOREF, M.F., TRICKER, K.J., PROSSER, J., CONDIE, A., KELSEY, A.M., HARRIS, M., MORRIS JONES, P.H., BINCHY, A., CROWTHER, D., CRAFT, A.W., EDEN, O.B., EVANS, D.G.R., THOMPSON, E., MANN, J.R. & MARTIN, J. (1994). Prevalence and diversity of constitutional mutations in the p53 gene among 21 Li-Fraumeni families. *Cancer Res.*, **54**, 1298–1304.

BRUGIÈRES, L., GARDES, M., MOUTOU, C., CHOMPRET, A., MERESSE, V., MARTIN, A., POISSON, N., FLAMANT, F., BONAÏTI-PELLIÉ, C., LEMERLE, J. & FEUNTEUN, J. (1993). Screening for germ line p53 mutations in children with malignant tumors and a family history of cancer. *Cancer Res.*, **53**, 452–455.

DITTMER, D., PATI, S., ZAMBETTI, G., CHU, S., TERESKY, A.K., MOORE, M., FINLAY, C. & LEVINE, A.J. (1993). Gain of function mutations in p53. *Nature Genet.*, **4**, 42–45.

EASTON, D.F., BISHOP, D.T., FORD, D., CROCKFORD, G.P. AND THE BREAST CANCER LINKAGE CONSORTIUM (1993). Genetic linkage analysis in familial breast and ovarian cancer: results from 214 families. *Am. J. Hum. Genet.*, **52**, 678–701.

FUTREAL, P.A., BARRETT, J.C. & WISEMAN, R.W. (1991). An Alu polymorphism intragenic to the TP53 gene. *Nucleic Acids Res.*, **19**, 6977.

GARBER, J.E., GOLDSTEIN, A.M., KANTOR, A.F., DREYFUS, M.G., FRAUMENI, Jr, J.F. & LI, F.P. (1991). Follow-up study of twenty-four families with Li–Fraumeni syndrome. *Cancer Res.*, **51**, 6094–6097.

HARRIS, N., BRILL, E., SHOHAT, O., PROKOCIMER, M., WOLF, D., ARAI, N. & ROTTER, V. (1986). Molecular basis for heterogeneity of the human p53 protein. *Mol. Cell. Biol.*, **6**, 4650–4656.

HEIGHWAY, J., THATCHER, N., CERNY, T. & HASLETON, P.S. (1986). Genetic predisposition to human lung cancer. *Br. J. Cancer*, **53**, 453–457.

HEIGHWAY, J., MITCHELL, E.L.D., JONES, D., WHITE, G.R.M. & SANTIBÁÑEZ-KOREF, M.F. (1994). A transcribed polymorphism and sub-localisation of MDM2. *Hum. Genet.*, **93**, 611–612.

JONES, M.H. & NAKAMURA, Y. (1992). Detection of loss of constitutional heterozygosity at the human TP53 locus using a dinucleotide repeat. *Genes Chrom. Cancer*, **5**, 89–90.

KONDOLEON, S., VISSING, H., LUO, X.Y., KELLOG, J. & LITT, M. (1987). A hypervariable RFLP on chromosome 17p13 is defined by an arbitrary single copy probe p144-D6. *Nucleic Acids Res.*, **15**, 10605.

LANE, D.P. & BENCHIMOL, S. (1990). p53: oncogene or anti-oncogene? *Genes Dev.*, **4**, 1–8.

LAW, J.C., STRONG, L.C., CHIDAMBARAM, A. & FERRELL, R.E. (1991). A germ line mutation in exon 5 of the p53 gene in an extended cancer family. *Cancer Res.*, **51**, 6385–6387.

LEACH, F.S., TOKINO, T., MELTZER, P., BURRELL, M., OLINER, J.D., SMITH, S., HILL, D.E., SIDRANSKY, D., KINZLER, K.W. & VOGELSTEIN, B. (1993). *p53* mutation and *MDM2* amplification in human soft tissue sarcomas. *Cancer Res.*, **53**, 2231–2234.

LUSTBADER, E.D., WILLIAMS, W.R., BONDY, M.L., STROM, S. & STRONG, L.C. (1992). Segregation analysis of cancer in families of childhood soft-tissue-sarcoma patients. *Am. J. Hum. Genet.*, **51**, 344–356.

MCGEE, T.L., COWLEY, G.S., YANDELL, D.W. & DRYJA, T.R. (1990). Detection of the XbaI RFLP within the retinoblastoma locus by PCR. *Nucleic Acids Res.*, **18**, 207.

MALKIN, D., LI, F.P., STRONG, L.C., FRAUMENI, Jr, J.F., NELSON, C.E., KIM, D.H., KASSEL, J., GRYKA, M.A., BISCHOFF, F.Z., TAINSKY, M.A. & FRIEND, S.H. (1990). Germ line p53 mutations in a familial syndrome of breast cancer, sarcomas, and other neoplasms. *Science*, **250**, 1233–1338.

MIDGLEY, C.A., FISHER, C.J., BARTEK, J., VOJTESEK, B., LANE, D.P. & BARNES, D.M. (1992). Analysis of p53 expression in human tumours: an antibody raised against human p53 expressed in *E. coli*. *J. Cell Sci.*, **101**, 183–189.

MOMAND, J., ZAMBETTI, G.P., OLSON, D.C., GEORGE, D. & LEVINE, A.J. (1992). The mdm-2 oncogene product forms a complex with the p53 protein and inhibits p53-mediated transactivation. *Cell*, **69**, 1237–1245.

OLINER, J.D., KINZLER, K.W., MELTZER, P.S., GEORGE, D.L. & VOGELSTEIN, B. (1992). Amplification of a gene encoding a p53-associated protein in human sarcoma. *Nature*, **358**, 80–83.

REIFENBERGER, G., LIU, L., ICHIMURA, K., SCHMIDT, E.E. & COLLINS, V.P. (1993). Amplification and overexpression of the (iMDM2) gene in a subset of human malignant gliomas without (ip53) mutations. *Cancer Res.*, **53**, 2736–2739.

SANTIBÁÑEZ-KOREF, M.F., BIRCH, J.M., HARTLEY, A.L., MORRIS JONES, P.H., CRAFT, A.W., EDEN, T., CROWTHER, D., KELSEY, A.M. & HARRIS, M. (1991). p53 germline mutations in Li–Fraumeni syndrome. *Lancet*, **338**, 1490–1491.

SRIVASTAVA, S., ZOU, Z., PIROLLO, K., BLATTNER, W. & CHANG, E.H. (1990). Germ-line transmission of a mutated p53 gene in a cancer-prone family with Li–Fraumeni syndrome. *Nature*, **348**, 747–749.

STRATTON, M.R., WILLIAMS, S., FISHER, C., BALL, A., WESTBURY, G., GUSTERSON, B.A., FLETCHER, C.D.M., KNIGHT, J.C., FUNG, Y.-K., REEVES, B.R. & COOPER, C.S. (1989). Structural alterations of the RB1 gene in human soft tissue tumours. *Br. J. Cancer*, **60**, 202–205.

TEARE, M.D., SANTIBÁÑEZ-KOREF, M.F., WALLACE, S.A., WHITE, G.R.M., EVANS, D.G.R., BURNELL, L.D., HARRIS, M., HOWELL, A. & BIRCH, J.M. (1993). A linkage study in seven breast cancer families. *Am. J. Hum. Genet.*, **52**, 786–788.

TOGUCHIDA, J., YAMAGUCHI, T., DAYTON, S.H., BEAUCHAMP, R.L., HERRERA, G.E., ISHIZAKI, K., KAMAMURO, T., MAYERS, P.A., LITTLE, J.B., SASAKI, M.A., WEICHSELBAUM, R.R. & YANDELL, D.W. (1992). Prevalence and spectrum of germline mutations of the p53 gene among patients with sarcoma. *N. Engl. J. Med.*, **326**, 1301–1308.